# Supplementary material for: Are carbon emissions trading and green financial instruments synergistic? -Comprehensive quantitative research based on content analysis
Source: PLoS One. 2024 Mar 7;19(3):e0298601. doi: 10.1371/journal.pone.0298601 (PMC10919601; doi:10.1371/journal.pone.0298601)
Supplement: S1 Data — (ZIP) [file pone.0298601.s001.zip › Data/地级市Z_F工作报告.zip!/╦─┤¿/─┌╜¡╩╨/└┤╫╘╣1⁄2╓┌║┼í╛┬φ┐╦╩2╛▌═°í┐_2019.docx]

各位代表：

　　我代表市人民政府，向大会报告工作，请予审议，并请市政协委员提出意见。

2018年工作回顾

　　2018年，在省委、省政府和市委的坚强领导下，在市人大、市政协的监督支持下，我们坚定以习近平新时代中国特色社会主义思想为指导，深入学习贯彻党的十九大精神和习近平总书记对四川工作系列重要指示精神，紧紧围绕市七届人大四次会议确定的目标任务，坚持稳中求进工作总基调，用心认真尽责，敢挺苦干实拼，经济社会保持平稳健康发展。地区生产总值增长7.8%；地方一般公共预算收入同口径增长8.9%；城乡居民人均可支配收入分别增长8.5%、9.3%。

　　一、深入学习贯彻习近平总书记对四川工作系列重要指示精神，认真落实省委、市委决策部署

　　我们把学习贯彻习近平总书记对四川工作系列重要指示精神作为重大政治任务，认真落实省委十一届三次、四次全会决策部署，深入开展“大学习、大讨论、大调研”活动，细化落实“一干多支、五区协同”“四向拓展、全域开放”战略部署，按照市委七届七次、八次全会安排，加快建设成渝发展主轴重要节点城市和成渝特大城市功能配套服务中心，开启内江高质量发展新征程。我们把产业作为发展的根基，瞄准成渝主导产业配套环节和服务需求，全面分析转型升级、高质量发展态势，着力构建“5+4+5”现代产业体系。“四新一大”产业实现总产值277.3亿元，占规模工业总产值的比重达26.1%；服务业增加值占地区生产总值的比重达41.2%，比2017年提高6.5个百分点；农业增加值增长3.8%，居全省第3位。我们以更大力度推进开放合作，成功引进香港新华国际金融中心、杭萧钢构装配式建筑等5亿元以上项目57个，签署区域合作协议34项，授牌设立川内首个“蓉欧+”东盟国际班列市（州）基地，“一点三线”立体全面开放新态势加快形成。我们聚力发展县域经济，大力实施“五个县市区+内江经开区+内江高新区”七轮驱动，县域竞相发展、协同发展新格局加快构建。我们全力激发市场活力、稳定发展信心，召开民营经济健康发展大会，大张旗鼓表彰优秀民营企业家，制定25条扶持政策措施，民间投资增长21.6%，内江正成为企业家成长的摇篮、返乡下乡创业者的乐园。

　　二、坚持把发展作为第一要务，经济保持稳定增长

　　工业效益稳中向好。规模以上工业增加值增长9.3%；工业入库税金增长15.8%；工业用电量增长20.7%，居全省第2位。大力推进先进制造强市“三百行动”，全年新发展先进制造企业41户，传统制造企业实现技改37户，培育制造业名优品牌36个，新增规模以上工业企业39户。冶金建材、机械汽配等传统优势产业加快转型，高新技术产业产值占规模工业总产值的比重达35.6%。支持企业实施智能化改造，浩物机电曲轴数字化生产线建成投用，巨腾国际“机器换人”项目完成建设。加大企业扶持力度，帮助企业降低用电、用气成本4.9亿元，提供融资担保19.5亿元，办理应急转贷资金8.3亿元。全市重点工业园区新增承载能力面积2.4平方公里，新入驻单项投资5000万元以上项目25个。

　　服务业稳步发展。服务业增加值首次突破500亿元，达到581.6亿元，增长8.7%。社会消费品零售总额增长10.9%。举办2018中国（四川）电子商务发展峰会，全年实现电商网络交易额644.4亿元、居全省第3位，快递业务量增长44.9%。实施旅游发展“三五八”方案，乐贤半岛旅游区成功创建国家4A级景区，全市实现旅游总收入311.7亿元，增长18.1%。新增金融机构（准金融机构）3家，全市各项存款余额1652亿元，增长8.9%；各项贷款余额902.8亿元，增长15.3%，较年初新增120亿元，创历年新高。

　　重点项目加快实施。狠抓项目投资，采取市级领导联系重点项目和定期举行项目集中开工活动等措施，实施33个省级、315个市级重点项目，分别完成投资42.7亿元、237.3亿元，102个重点项目开工建设，30个重点项目竣工投用，完成全社会固定资产投资716.7亿元。内江城市过境高速公路、成宜高速公路、银山渡改公路桥等重点项目有序推进，川南城际铁路、连乐铁路加快建设，内江综合客运中心站顺利竣工，新（改）建农村公路1033公里，完成交通建设投资31.2亿元。长征渠引水工程规划修编正式启动，向家坝灌区北总干渠一期工程开工建设，东兴区联合水库大坝枢纽全面完工。

　　三、坚决打好三大攻坚战，决胜全面小康的基础更加坚实

　　脱贫攻坚连战连捷。坚持把脱贫攻坚作为最大的政治责任、最大的民生工程、最大的发展机遇，投入各类扶贫资金21.3亿元，实施22个扶贫专项，完成易地扶贫搬迁3139户、8516人，改造贫困户危房5667户，实现88个贫困村退出、25366名贫困群众脱贫。3063名干部到农村结对帮扶，脱贫攻坚力量不断壮大。积极响应省委号召，选派188名干部综合帮扶凉山州精准脱贫。

　　污染防治扎实推进。大力实施全国首批流域水环境综合治理与可持续发展试点，获批全国城市黑臭水体治理示范城市，省级森林城市通过考核验收。认真执行《内江市甜城湖保护条例》，打好污染防治“八大战役”，沱江流域水环境综合治理、城乡生活垃圾处理2个PPP项目开工建设。扎实抓好中央、省级环保督察及“回头看”反馈问题整改，编制“1+6+N”整改方案，解决突出环境问题970个。中心城区PM10、PM2.5年平均浓度分别下降16.5%、20.4%，降幅均排全省第2位，空气优良天数率提高6.9个百分点，全年达到297天。河（湖）长制全面落实，沱江干流内江出境断面达到Ⅲ类水质，球溪河球溪河口断面、威远河廖家堰断面达到Ⅳ类水质。土壤环境质量总体稳定。

　　有效防范化解各类风险。认真落实9个防范化解重大风险工作方案，积极防范化解各领域风险。严厉打击非法集资，妥善处理企业债务，全市银行业金融机构不良贷款率下降2.2个百分点。动态监控政府债务增减变动情况，政府债务总体可控。狠抓重点行业领域安全隐患排查整改，全市各类生产安全事故发生起数、死亡人数分别下降31.7%、17.9%。持续推进“明厨亮灶”建设，连续14年未发生重特大食品药品安全事故。沉着应对“7·12”沱江内江段37年来最大洪峰过境，实现“零死亡、零失踪、零重伤”。全力打好非洲猪瘟防控战，内江境内无疫情发生。

　　四、推进以人为核心的新型城镇化，城乡面貌逐步改善

　　“双百”城市扩容提质。坚持规划引领，编制完成城市绿地系统、地下综合管廊、基础教育设施等12个专项规划，以及邓家坝片区、谢家河片区等4个片区（组团）控制性详规和5项城市设计。实施“双百”城市项目205个，中心城区建成区面积达90.2平方公里、常住人口达90.5万人。骨干路网加快完善，打通北环线一至四段，新建沱江大桥建成通车，邱家嘴立交桥南延线与石羊大道实现贯通。完善公共配套设施，高铁站前综合配套项目等14个项目完工，一批公共地下停车场、充电站（桩）、加油站投入使用。深入实施“城市变景区”工程，谢家河公园北段、玉峰山公园、千水湖公园建成并向市民开放。国家卫生城市创建工作稳步推进，开展城乡结合部、背街小巷、老旧院落整治，搬迁河坝街旧家具市场、红牌路蔬菜批发市场。强力拆除违法建设6865处、60余万平方米，改造危旧房棚户区7774套，城市环境明显改善。

　　乡村振兴全面启动。统筹推进“五个振兴”，扎实抓好“十二件事”，粮食产量实现“十二连增”。编制出台《内江市乡村振兴战略规划（2018—2022年）》，启动3个省级、24个市级乡村振兴规划试点编制工作。深入实施100万头“内江黑猪”产业化项目，祖代种猪场建设完工；新建稻渔综合种养产业带13万亩，建成优质粮油、精品农业、循环农业、创意体验农业功能区17个，集中连片万亩特色产业基地达18个，建成3.2万亩全国最大的无花果全产业链基地、24万亩全国最大的血橙生产基地，国家农业科技园区通过科技部复核验收。新增省级农业产业化龙头企业5户，发展农民专业合作社335个、家庭农场351个。作为全省唯一主题市参加第十届全国农产品展销周活动，“甜城味”优质农产品品牌影响力进一步扩大。启动实施人居环境整治三年行动，建成幸福美丽新村200个，创建省级、市级“四好村”90个、255个。实施农村饮水安全巩固提升工程，整改水质不合格水井1161处，新增受益人口5.1万人。

　　五、加大改革开放力度，发展活力持续增强

　　重点领域改革深入推进。以实际行动纪念改革开放40周年，完成国省改革试点任务15项，稳步推进公务员职务与职级并行等全国改革试点。深化商事制度改革，新登记市场主体2.5万户。统筹推进政府机构、供销社、国资国企等重点领域和关键环节改革。深化全面创新改革，新建省级科技企业孵化器1个、省级工程技术研究中心3个，与清华大学深圳研究生院、机械科学研究总院集团等5所高等院校、科研院所签署战略合作协议，隆昌市成功入选全国首批创新型县（市）建设名单。

　　全面开放态势加速形成。积极参加“中外知名企业四川行”、第十七届西博会、“川港澳合作周”等活动，举办第九届大千龙舟经贸文化节等本土活动，赴上海、广东、浙江、香港等地开展投资促进活动。招商引资到位市外资金540.2亿元，增长12.5%。深入实施返乡下乡创业“回家工程”，新增返乡下乡创业人员1563人，创办企业229户，总投资45.7亿元。组团参加首届中国国际进口博览会，“内江造”柴油机、玻璃纤维制品、羽绒制品远销海外，进出口总额增长47.6%、居全省第7位，引进外资增长62.5%，开放型经济发展水平实现跃升。

　　区域合作迈出坚实步伐。深入对接成都“东进”战略，举办成都·内江深化合作座谈会暨协同发展重大项目签约仪式，签署基础设施、产业协同、文化旅游等方面专项协议26项，已生成重点项目12个、总投资69亿元。明确与重庆协同发展69项任务，与荣昌签署《推进区域协同发展战略合作协议》，明确共建川渝合作荣隆工业园。制定内江推进川南经济区一体化发展五年行动计划，与川南三市签署合作协议7项。启动编制内自同城化发展规划，内自同城化迈出实质性步伐。

　　六、践行以人民为中心的发展思想，人民生活持续改善

　　民生工程全面完成。“十项民生工程”“20件民生实事”和民生“十有行动”目标任务顺利完成，民生支出160.5亿元，占一般公共预算支出的65.9%，高于全省平均水平0.8个百分点。实现城镇新增就业5.4万人。为2.2万名义务教育家庭经济困难寄宿制学生发放生活补助，为3.8万名农村义务教育在校学生提供免费营养午餐。开通省内医保异地结算医院38家，我市成功创建全国基层中医药工作先进单位。继续为困难家庭失能老人提供居家养老服务，向80周岁以上老人发放高龄补贴，改造农村公办适老化床位1512张。15.6万残疾人享受到“量体裁衣”式服务。新（改）建标准化农贸市场（便民服务点）29个、公共厕所244座。

　　社会事业协调发展。市第一人民医院新区、内江六中高新校区等项目投入使用，内江卫生与健康职业学院、市文化馆、大千园综合提升改造工程等项目基本完成，市体育中心、市第二人民医院门诊综合楼、内江师院新校区等项目加快推进。在全省率先出台加快幼儿园建设发展实施方案，新（改、扩）建公办幼儿园15所，全面实现县域义务教育均衡发展，全市本科上线人数突破万人大关。完成34个行政村广播“村村响”和1万户电视“户户通”建设。大力弘扬社会主义核心价值观，持续开展“践行十爱·德耀甜城”主题活动。资中木偶剧团被表彰为全国服务农民、服务基层文化建设先进集体，内江川剧灯戏入围国家艺术基金年度资助项目。承办省级以上大型体育赛事7场次，开展“周末快乐体育”活动800余场次，内江代表团在省第十三届运动会上获得奖牌92枚，继续保持全省领先地位。

　　社会保持和谐稳定。新（改）建城区派出所11个，市公安局交通安全指挥中心建成投用。深化“七五”普法，新建乡（镇、街道）规范化法律援助工作站3个，市、县级综治中心建成投用。深入开展扫黑除恶专项斗争，全市刑事案件下降10%。积极化解信访维稳突出问题，群众合法权益得到有效维护。国家安全、国防教育、国防动员、人民防空、双拥共建、优抚安置等工作取得新进步。外事侨务、对台工作、民族宗教、统计调查、气象水文、防灾减灾、海关检验检疫、无线电管理、保密、档案、史志等工作实现新发展。新闻出版、红十字、慈善、老龄、妇女儿童等事业取得新成就。

　　七、持续转变工作作风，政府自身建设全面加强

　　自觉接受市人大、市政协监督，办理人大代表议案1件、建议批评意见99件，办理政协委员提案270件，办结率100%。主动接受监察监督，广泛听取民主党派、工商联、无党派人士、人民团体的意见，积极支持工会、共青团、妇联等群团组织开展工作。严格依法行政，提请审议地方性法规2件，对260余件次文件、协议进行合法性审查。公布“最多跑一次”事项1295项，企业开办时间压减至5个工作日。持续深化政务公开，主动公开政府信息14.8万条。严格公务用房、公务用车管理，财政拨款“三公”经费下降9.2%。扎实推进党风廉政建设，加强审计工作，持续深化扶贫领域腐败和作风问题专项治理，惠民惠农财政补贴资金“一卡通”管理全省试点工作取得阶段性成效。

　　各位代表，过去一年的成绩来之不易。这是市委坚强领导、团结带领全市人民真抓实干、拼搏奋斗的结果，是市人大、市政协和社会各界有效监督、大力支持的结果。在此，我谨代表市人民政府，向420万内江人民，向人大代表、政协委员、各民主党派、工商联、无党派人士、各人民团体，向驻内部队、武警官兵、政法干警和民兵预备役人员，向关心、支持、参与内江建设发展的各界朋友，表示诚挚的感谢！

　　在看到成绩的同时，我们也清醒认识到，内江经济社会发展中还存在一些困难和问题，服务业增加值增速、建筑业增加值增速、全社会固定资产投资增速、社会消费品零售总额增速、城镇化率等未能实现预期目标。产业结构不优，支柱产业不强，项目支撑不足，先进制造业和现代服务业发展还不充分。实体经济运行成本较高，部分企业生产经营困难。财力基础薄弱，税源亟待培育。城乡发展还不平衡，县域经济活力不强，乡村振兴任重道远。保障和改善民生任务繁重，城市交通拥堵还未根本缓解，教育医疗、饮水安全等与群众期盼还有较大差距。干部队伍能力素质有待进一步提高，营商环境仍需优化。我们将直面问题、勇于担当、扎实工作，努力开创工作新局面。

2019年重点工作安排建议

　　2019年是新中国成立70周年，是深入学习贯彻习近平新时代中国特色社会主义思想和党的十九大精神的重要一年，是决胜全面建成小康社会的关键之年。今年政府工作的总体要求是：以习近平新时代中国特色社会主义思想为指导，全面贯彻党的十九大和中央经济工作会议精神，认真落实省委十一届三次、四次全会和省委经济工作会议精神，按照市委七届七次、八次全会和市委经济工作会议部署，统筹推进“五位一体”总体布局，协调推进“四个全面”战略布局，坚持稳中求进工作总基调，坚持新发展理念，坚持推动高质量发展，坚持以供给侧结构性改革为主线，坚持深化市场化改革、扩大高水平开放，大力实施“产业项目重点推进年”部署，着力构建“5+4+5”现代产业体系，继续打好“三大攻坚战”，统筹推进稳增长、促改革、调结构、惠民生、防风险各项工作，进一步稳就业、稳金融、稳外贸、稳外资、稳投资、稳预期，优化营商环境，提振市场信心，增强人民群众获得感幸福感安全感，保持经济持续健康发展和社会大局稳定，为全面建成小康社会收官打下决定性基础，以优异成绩庆祝中华人民共和国成立70周年。

　　今年全市经济社会发展的主要预期目标是：地区生产总值增长7.8%左右；全社会固定资产投资增长10%；规模工业增加值增长9.1%；社会消费品零售总额增长11.5%；地方一般公共预算收入增长与经济增长基本同步；城乡居民人均可支配收入分别增长8.5%、9%；城镇登记失业率控制在4.5%以内；居民消费价格总水平涨幅控制在3%以内；完成省下达的节能减排和环境保护目标任务。

　　提出以上目标，总体是积极的，既充分考虑了内江发展实际，对接了经济高质量发展的总体要求，又充分考虑了与全国全省同步实现全面小康、引导市场预期和增加就业的需要，以及内江项目投资、产业发展等方面的支撑条件，符合稳中求进工作总基调，是必须经过艰苦努力、跳起摸高才能实现的。

　　围绕目标，重点抓好以下工作。

　　一、持续打好三大攻坚战

　　打好精准脱贫攻坚战。全面落实打赢精准脱贫攻坚战三年行动实施方案，实现存量的65个贫困村全部退出、15064名贫困群众全部脱贫。深入实施19个扶贫专项，建立“四项扶贫基金”县级补充长效机制。抓好易地扶贫搬迁后续工作，加大产业扶持和就业帮扶力度。大力实施“创业致富带头人”“农村家庭能人”培养计划，扎实开展“回头看”“回头帮”，减少和防止已脱贫群众返贫。对照脱贫标准，全面开展“查问题、找不足、补短板”活动。研究解决收入水平略高于建档立卡贫困户的群体缺乏政策支持等新问题。

　　打好污染防治攻坚战。大力实施“1+6+N”整改方案，全面开工沱江流域水环境综合治理、城乡生活垃圾处理PPP项目152个子项目，确保完工90个以上。持续打好污染防治“八大战役”，实施“五大工程”，常态化开展“五清”行动，建成市第二污水处理厂、市生活垃圾处理中心，完成市第一污水处理厂等5座城市污水处理厂提升改造，中心城区空气优良天数率超过75.5%。完成第二次全国污染源普查工作，开展乡镇集中式饮用水水源地环境问题排查整治，强化土壤环境风险防控。

　　打好防范化解重大风险攻坚战。坚持底线思维，严格落实保安全、护稳定各项措施，严守社会稳定、安全生产、食品药品安全底线，做好防汛抗旱和森林防火工作。严厉打击非法集资，推进互联网金融风险专项整治，防范化解企业债务风险，实现全市银行业金融机构不良贷款率下降1个百分点。强化政府债务管理，坚决遏制隐性债务增量。加强房地产市场监管，促进房地产市场平稳健康发展。

　　二、实施“产业项目重点推进年”

　　着力扩大有效投资。落实领导干部联系重点项目制度，坚持按月调度，实施省、市重点项目30个、425个，分别完成投资61.8亿元、274.7亿元。围绕“四新一大”产业，谋划实施一批重点产业项目，技改投资增长10%以上。围绕全域开放、区域合作，加快谋划涉及国际陆海贸易新通道、川南一体化等领域重大项目，完成交通基础设施投资20亿元以上。围绕绿色发展，包装实施一批环保装备、污染治理、循环经济项目。围绕民生改善，布局一批教育、文化、卫生等项目。围绕乡村振兴，谋划实施一批农业现代化项目，加快推进向家坝灌区北总干渠一期、资中县两河口水库、威远县大石包水库等水利工程建设，力争将全市可受益灌溉面积和沱江生态补水整体纳入长征渠规划。围绕脱贫攻坚，包装实施一批产业扶贫、电商扶贫项目。大力建设标准厂房，提高投资强度，新增工业园区承载能力面积2平方公里。探索多渠道投融资模式，确保民间投资稳步增长。

　　推动制造业高质量发展。继续实施先进制造强市“三百行动”，确保发展先进制造企业40户以上、技改传统制造企业40户以上、培育制造业名优品牌40个以上，新增规模工业企业40户以上。大力发展“四新一大”产业，占规模工业总产值的比重提高2个百分点。新材料产业重点推进川威五氧化二钒扩能、智能炼钢等项目，加快高新区白马新材料产业园建设。新装备产业重点实施福伊尔动力高端曲轴生产线、山川精密焊管长安汽车构件产业园等项目。新医药产业重点加强药物研发和生产，大力发展中医药产业。新能源产业重点推进内江新能源汽车产业园建设，引进相关配套产业，加快充电站（桩）等基础设施建设，推进页岩气勘探开发和就地转化利用。大数据产业重点抓好智能宽带、信息安全产业园建设，全面拥抱新经济，提升优质生活体验，突破地域发展瓶颈，打造“数字内江”。

　　推动服务业提档升级。积极参加市场拓展“三大活动”，组织开展“内江产品专场”促销活动，稳住汽车、家电等热点产品消费，扩大教育培训、文化体育等消费，推动消费升级。分类实施服务业企业培育计划，支持个体工商户转型升级为企业，新增限额以上商贸企业和规模以上服务业企业30户。加快建设7大电商集聚区、8大电商平台，推进线上线下互动融合，实现电商网络交易额740亿元，增长15%以上。大力发展文化旅游产业，实施全域旅游战略，加快推进罗泉古镇、石板河旅游区等20个重点项目建设，力争新创建国家4A级景区或省级旅游度假区2个，旅游总收入增长12%以上。积极发展现代物流业，加快建设“蓉欧+”东盟国际物流中心。挖掘康养产业潜力，不断加大优质养老服务产品供给。加快发展金融业，实施多层次资本市场培育计划，新增金融机构（准金融机构）2家以上。

　　三、构建“一点三线”立体全面开放新态势

　　高标准建设开放平台。积极参加中国西部（四川）国际投资大会、“中外知名企业四川行”等重大活动，办好“国企内江行”、大千龙舟经贸文化节、川南电商博览会等活动，加快引进一批优质项目。着力完善开放平台，加快建设自贸试验区协同改革先行区和保税物流中心（B型），完成内江海关建设，力争进出口贸易总额增长32%以上。

　　加快建设开放通道。深入实施“一带一轴一区”重要交通枢纽建设三年推进行动，建成内江城市过境高速公路，加快推进川南城际铁路、连乐铁路、成宜高速公路等项目，开工建设成自高铁（内江段）等项目，力争开工建设自隆高速连接线（威远段），做好井研经资中至铜梁高速、乐至经资中至犍为高速、绵遂内城际铁路、隆黄铁路（隆昌至叙永段）等项目前期工作，争取天府机场经内江至大足高速公路纳入省高速公路网。

　　加大招商引资力度。整合招商力量，强化精准招商、延链招商、以企招商，确保到位市外资金增长13%以上。面向沿海、粤港澳大湾区，积极承接优质产业转移；面向德国、日本、韩国等发达国家，加快引进汽车制造、电子信息等产业，力争吸引一批配套项目落户内江；面向台湾地区，发挥大千文化纽带作用，发展特色台资产业园区，争取建立国家级“海峡两岸青年创业基地”；面向东盟、南亚，建立贸易进出口、留学生教育基地。扎实推进返乡下乡创业“回家工程”，新增返乡下乡创业人员1200人。

　　深化区域协同发展。推进与成都、重庆协同发展，落实好与成都协同发展系列合作协议。落实川南经济区一体化发展五年行动计划和年度重点工作方案，组建川南担保公司及相关产业联盟。充分用好内自同城化工作机制，编制完成内自同城化发展规划，适时启动内自快速通道建设，年内实现公积金贷款、异地就医结算、社保关系转移等公共服务同城化，推动内自同城化发展取得更大突破。

　　四、始终坚持改革创新

　　深化供给侧结构性改革。落实“巩固、增强、提升、畅通”八字方针，坚持去产能与优产能相结合，稳妥处置“僵尸企业”。落实直购电、富余电量等政策，推动天然气转供变直供，降低企业生产经营成本。健全多主体供给、多渠道保障、租购并举的住房制度，大力发展住房租赁市场。

　　统筹推进各项改革。加快经济体制改革，推进国企混合所有制改革，巩固农村集体产权制度改革成果。深化商事制度改革，新发展市场主体2.2万户。完成政府机构改革任务。持续抓好供销社、电力体制、财税体制等各领域改革。

　　深入实施创新驱动发展战略。加快建设区域性科技创新中心和创新驱动发展先行市，全力建设院士（专家）工作站、重点实验室、企业技术中心、科技企业孵化器等创新载体。深化产学研合作体系建设，新签订一批市校、校企合作协议，实施市级以上重大科技成果转化项目20项以上。举办第三届“内江高新杯”创客大赛等活动，打造“双创”区域示范城市。

　　五、扎实推进乡村振兴和县域经济发展

　　推动农村产业振兴。坚持藏粮于地、藏粮于技，实施“12345”现代农业产业提升行动和“351”特色农业产业发展方案，积极发展“三品一标”产品，加快建设农业产业孵化园，创建省级、市级现代农业园区7个以上，实现农业增加值增长3.8%。实施100万头“内江黑猪”产业化项目，建设家庭农场110个单元。实施资中血橙“双3计划·百亿产业”，建设血橙特色小镇1个。推动威远无花果产业提档升级，打造无花果博览园。提升“资中鲶鱼”“内江稻花鱼”“永安白乌鱼”等特色水产品牌影响力，继续推进30万亩稻渔综合种养产业带建设。

　　狠抓宜居乡村建设。高标准建设“美丽内江·宜居乡村”，开展农村人居环境整治，推进垃圾、污水、厕所“三大革命”，抓好农业面源污染治理等重点工作，力争90%以上的行政村生活垃圾和30%以上的行政村生活污水得到有效处理。全力推进“四好农村路”建设，打通一批乡村断头路。继续实施农村饮水安全巩固提升工程，加快农网改造升级。加强传统村落保护利用，培育创建一批“四川最美古村落”。

　　加快发展县域经济。支持各县（市、区）根据资源禀赋、发展基础和功能定位，完善基础设施和公共服务设施，促进产城融合、城乡共进。进一步完善推动县域经济高质量发展的支持政策和考核办法，强化分类指导和奖励激励。积极培育县域经济强县和生态示范标杆县，力争1—2个县（市、区）进入全省首批30个天府旅游名县候选名录。深化拓展“百镇建设行动”，持续抓好17个国、省重点镇建设，力争新创建1个省级特色小镇。

　　六、加快“双百”城市建设与发展

　　拓展城市空间。围绕“一中心四组团”布局，完成城市片区控制性详细规划，教育设施、养老设施等7个专项规划编制。推进组团间互连互通，做好成渝、内宜高速公路城区段置换前期工作，加快推进内江城市过境高速椑木东、高桥互通连接线建设，力争黄荆坝大桥及连接线建成通车，实现汉渝大道全线贯通，打通10条城市断头路。有序推进邓家坝片区整体开发，启动顺江路、迎霞路、康宁路等主干道路建设；推动谢家河片区、高铁片区、城南新区、白马组团、东兴南部新城、椑木新城提质扩面，推动内江经开区和内江高新区建设提速，确保中心城区建成区面积达到95平方公里、常住人口达到95万人。

　　完善城市功能。按照“城市双修”理念，继续实施城中村、老旧院落、背街小巷改造提升工程，重点推进四方块A地块、大冲山片区、胜利—高桥街道片区等危旧房棚户区改造，完成改造8438户。着力建设公园城市，加快银杏街绿廊、花萼湿地公园建设，建成谢家河湿地公园，完成人民公园提升改造。建设公共停车场5个，建成CNG加气站2个。

　　加强城市管理。以智慧城市建设为引领，巩固城市管理成效。狠抓“治堵”，启动城市智能交通系统建设。狠抓“治乱”，常态化整治占道经营、乱停乱放等问题，规范设置临时摊区。狠抓“治违”，强化联合执法，加大城区违法建设整治力度。狠抓“治脏”，排查整治污水管网，坚决防止污水直排，提升改造城区垃圾收集点，实现城区生活垃圾无害化处理率98%以上。全力创建国家卫生城市、国家园林城市、省级文明城市，提升城市知名度和美誉度。

　　七、推动民营经济健康发展

　　开展政策大落实行动。全面落实“内江民营经济25条”，把政策落实情况纳入政府激励考核，引导民营企业家坚定信心、安心发展。深入实施“311”干部帮扶基层行动，为民营企业排忧解难，引导金融机构加大扶持力度，着力解决民营企业融资难融资贵问题。开展产权保护、市场准入等领域专项督查，打通政策落地“最后一公里”。

　　支持民营企业转型发展。弘扬诚实守信、勤劳致富和企业家创业精神，引导民营企业依据自身条件和市场需求开展生产经营活动，优化企业法人治理结构和股权结构。实施民营企业“内江品牌”培育计划，支持民营企业推进产品创新、技术创新、商业模式创新。设立民营经济发展专项资金，支持民营企业实施技术改造，引导中小微企业向“专精特新”发展，培育更多行业“小巨人”企业。

　　构建亲清新型政商关系。全面实施市场准入负面清单制度和公平竞争审查制度，启用四川（内江）企业维权服务平台，畅通民营企业诉求渠道，依法保护企业家财产权、创新权益和自主经营权。支持成立行业协会商会和产业联盟，开展民营企业评议政府部门活动，在全社会营造尊商、重商、亲商、安商良好氛围。

　　八、持续抓好生态文明建设

　　扎实推进沱江流域综合治理。深入开展沱江流域（内江段）水环境综合治理与可持续发展国家试点，编制实施沱江流域文化生态带点线面发展与保护空间布局规划。抓好城市黑臭水体治理示范城市建设，完成城区11条黑臭水体整治。全面落实河（湖）长制，开展全流域协同治理，加大球溪河、威远河、隆昌河等主要河流治理力度，确保沱江干流内江出境断面保持Ⅲ类水质，球溪河球溪河口断面、威远河廖家堰断面水质达到省考核要求。

　　统筹山水林田湖草系统治理。落实主体功能区规划，严守生态红线，提升生态系统自我修复功能。实施大规模绿化内江行动，完成营造林10万亩。贯彻实施《内江市城市园林绿化条例》。加强水土流失综合治理，强化湿地保护，恢复国土生态功能。开展“大棚房”问题和重点区域违规违法占用国家资源专项清理整治。

　　推动形成绿色生产生活方式。发展壮大节能环保、清洁能源等产业，推进资源全面节约和循环利用，促进产业布局与资源环境承载能力相适应。倡导简约适度、绿色低碳的生活方式，开展绿色家庭、绿色学校、绿色社区等示范创建，增强全民环保意识。

　　九、全力保障和改善民生

　　抓好民生工程。抓实“30件民生实事”，推进民生“十有行动”，确保民生支出占一般公共预算支出比例不低于全省平均水平。加强高校毕业生、农民工等群体就业创业服务，启动人力资源综合服务中心建设，确保新增城镇就业4万人。继续为义务教育家庭经济困难寄宿制学生发放生活补助，为4.4万名农村义务教育在校学生提供免费营养午餐。深入实施全民参保计划，加强城乡养老服务体系建设。抓好最低生活保障与特困人员供养、临时救助、残疾人帮扶等工作。加快推进谢家河西侧安置还房建设，确保被征地农民按时入住。改造农村危旧房6689户，治理地质灾害隐患点3处，避险搬迁安置120户。

　　办好社会事业。加快发展学前教育，新增标准幼儿园20所；推进义务教育优质均衡发展，积极化解大班额；抓好高考综合改革，启动内江一中改（扩）建等项目；促进职业教育产教融合、高等教育内涵式发展，加快内江师院新校区、内江职院、川南幼专后期工程建设，确保内江卫生与健康职业学院高校设置通过评审；办好特殊教育，支持民办教育健康发展。推进健康内江建设，深化公立医院综合改革，完善分级诊疗制度，加强重大疾病防控，加快市妇幼保健院业务用房等项目建设。加大历史文化保护力度，抓好重大文化惠民活动和精品文艺创作，开工建设内江大剧院，办好张大千诞辰120周年、范长江诞辰110周年等纪念活动。加快市体育中心建设，大力倡导全民健身，提升竞技体育水平。完成第四次全国经济普查。稳步推进退役军人移交安置和就业创业工作，健全退役军人服务管理和保障体系。扎实做好国防动员、民族宗教、外事侨务、对台工作、友协、保密、档案、史志等工作，大力发展妇女儿童、老龄、红十字、广播电视和慈善等事业。

　　强化社会治理。深化“平安内江”建设，纵深推进扫黑除恶专项斗争，坚决打击盗抢骗等侵财犯罪和经济领域违法犯罪。打好禁毒人民战争，启动特殊人群康复中心建设。推进社会治理信息化、智能化，加快建设“雪亮工程”“天网工程”。坚持发展新时代“枫桥经验”，健全公共法律服务体系和矛盾纠纷多元化解机制。完善应急管理机制，加强应急救援队伍建设。

努力打造人民满意政府

　　新形势新任务对政府工作提出更高要求，我们将坚定以习近平新时代中国特色社会主义思想为指导，增强“四个意识”、坚定“四个自信”、坚决做到“两个维护”，加强政府系统党的建设，加强政府职能转变和作风建设，努力建设人民满意的服务型政府。

　　着力建设法治政府。全面推进依法行政，不断提高政府公信力。认真执行人大及其常委会的决议决定，主动接受人大工作监督和法律监督、政协民主监督，接受监察监督，支持司法监督。坚持政府常务会会前学法，完善法律顾问制度，提高依法行政本领。严格落实重大行政决策的公众参与、专家论证、风险评估、合法性审查、集体讨论决定等法定程序。全面实行行政执法持证上岗，严格规范执法行为。全面推行政务公开，加强政策解读，回应社会关切。有序推进地方立法，加快制定地方性政府规章。

　　着力建设效能政府。深化“放管服”改革和“互联网+政务服务”，开展“营商环境提升年”活动。全面推行审批服务“马上办、网上办、就近办、一次办”，努力实现群众和企业办事“最多跑一次”，推动项目审批提速。完善重大事项督办机制，集中攻坚一批重点难点事项。强化监督问责，坚决整治不作为、慢作为、乱作为。健全正向激励、容错纠错、考核评价、关心关爱等机制，调动广大干部积极性。既立足现有条件，又瞄准高端、对标前沿，用新理念研究新情况，用新办法解决新难题，努力创造更多在全国、全省有影响力的工作业绩。

　　着力建设廉洁政府。坚持党风廉政建设“一岗双责”责任制，深入开展蒲波严重违纪违法案警示教育和“以案促改”工作，营造不敢腐、不能腐、不想腐的氛围。建设节约型机关，按照不低于5%的幅度压减一般性支出。加强审计监督，确保重大工程、扶贫项目、环保整改等重点领域审计监督全覆盖。认真落实中央八项规定精神，集中整治形式主义、官僚主义，坚决整治群众身边的不正之风和腐败问题，让人民群众感受到清风正气。

　　各位代表，让我们更加紧密地团结在以习近平同志为核心的党中央周围，在省委、省政府和市委的坚强领导下，紧紧依靠全市人民，迎难而上、开拓创新，奋力谱写新时代治蜀兴川内江实践新篇章，以优异成绩庆祝中华人民共和国成立70周年！
